# Pharmacy services during COVID-19 pandemic: experience from a tertiary care teaching hospital in Pakistan

**DOI:** 10.1186/s40545-020-00277-5

**Published:** 2020-11-02

**Authors:** Kashif Hussain, Gul Ambreen, Mehreen Muzammil, Syed Shamim Raza, Umer Ali

**Affiliations:** Department of Pharmacy, Aga Khan University Hospital, Stadium Road (Main Pharmacy), P.O Box 3500, Karachi, 74800 Pakistan

**Keywords:** Hospital pharmacy services, COVID-19, Pharmacist, Pharmaceutical care

## Abstract

The coronavirus disease 19 (COVID-19) is rapidly spreading across the world. Pharmacy services play a vital role in public health in preventing and containing the COVID-19 pandemic. All over the world, especially in the developed countries pharmacists have responded smartly and speedily for public health, such as establishing professional protective and service guidance for pharmacy staff and services, creating and updating drug formularies, addressing the issues of drug shortages, providing public education for prevention and management of infection, contributing in drug evaluation and clinical trials. In this commentary, we review the exclusive demands from pharmacy services in Pakistan during coronavirus disease 2019 pandemic and sharing the responses of our hospital pharmacy to these demands and needs with the international pharmacy community, especially of the low and middle-income countries like Pakistan.

## Background

After the outbreak of coronavirus disease 2019 (COVID-19) in China in December 2019, it spread all over the world. Novel COVID-19 is caused by severe acute respiratory syndrome coronavirus (SARS-CoV-2) [1]. The SARS-CoV-2 is different from human SARS CoV and Middle Eastern respiratory syndrome (MERS) CoV with respect to genetic characteristics [2]. Fever, shortness of breath, cough, breathing difficulties, and respiratory symptoms are the common signs of this infection. Pneumonia, severe acute respiratory distress syndrome, organ failure, and death are possible complications [1]. This dangerously infectious virus spreads rapidly through human-to-human transmission. The first case reported on 26 February 2020, in Pakistan. As of 19 July, there are 263,496 confirmed cases and 5568 deaths with coronavirus disease in Pakistan (available on: https://covid.gov.pk/stats/pakistan).

In low middle-income countries, like Pakistan, medical services are under great pressure while responding to this pandemic. In a tertiary care hospital pharmacy, along with the other approaches and responses in pandemic crises such as supply of emergency medications according to the treatment guidelines and resolving drug shortage, it is also required to provide and continue the event-driven pharmaceutical care. After the rapid transmission and influx of COVID-19 patients in our hospital, pharmacy department has adopted the national guidelines and modified the activities to reduce human-to-human infections transmission. All the modifications in the pharmacy activities based on the theme to provide event-driven pharmaceutical care with the least interaction and medication wastage caused by exposure to COVID-19 positive patient areas. Here we are sharing our response to COVID-19 pandemic and provision of inpatient hospital pharmacy services in a tertiary care hospital, Karachi, Pakistan to disseminate to the international pharmacy society what we are practicing despite being in the resource-limited region of the world.

## Response 1: providing event-driven pharmaceutical care

Pharmacists ensure medication safety, assist health care team to have best therapeutic outcome. The clinical pharmacy team is valued for directly making the difference for best possible outcome. Since 26th February 2020, when the first COVID-19 case was reported in Pakistan [3], health and health care systems have been disrupted extensively and most of the countries are still facing and fighting against the immediate consequences of higher mortality and morbidity rate due to severe acute respiratory syndrome coronavirus (SARS-CoV-2) [3]. Improving patient safety and work quality involve coordinated efforts of multidisciplinary health care team, including clinical pharmacist as the vital team member. In this evolving situation where the COVID-19 patients needed new therapies and experimental drugs been used for them, the need for a clinical pharmacist was more than ever before. To combat this challenging situation clinical pharmacy lead redesigned the front-line clinical pharmacist activities and job responsibilities. In normal routine our clinical pharmacists are involved in all the critical care wards of adult and pediatric units, where they participate in daily patient bedside rounds with the health care team. After the outbreak of COVID-19 and influx of infected patients, national guidelines were developed [3], and to stop the human-to-human transmission of infections minimum staff were allowed to be involved, isolation wards and rooms were created and allocated for COVID-19 patients [4]. With these fears and limitations, clinical pharmacy lead planned to provide event-driven pharmaceutical care in our hospital both for adult and pediatric COVID-19 patients. These practices go beyond the routine activities of the clinical pharmacist, including patient medications profile review, prescribing supports to physicians, and counseling the patients. The most important aspect of the event-driven pharmaceutical care is to ensure the usage of off-label drugs appropriately, as several drugs are prescribed off-label for treating this life-threatening infection [5]. The role of clinical pharmacist demands to evaluate the recent literature and published guidelines of these off-label used medications as the associated adverse drug reactions (ADRs) of few of the off-label used drugs include flu-like symptoms, fever, and fatigue, which are actual symptoms of the COVID-19 disease. Liver damage is reported in COVID-19 patients [6], which might be related to SARS-CoV-2 or drug-induced. Newly developed kidney damage has been reported in renal histopathological analysis of COVID-19 patients’ postmortem findings [7]. Here the role of the clinical pharmacist is very crucial to save the lives through monitoring the patients on drugs which may cause hepatoxicity, renal toxicity, or any other ADR and finally adjust the doses based on organ functionality.

## Response 2: tele-clinical pharmacist activities for making individualized treatment strategies with multidisciplinary team

Activities redesigned and now clinical pharmacists are doing round virtually specifically in COVID-19 unit. Distinctive challenges may be experienced while physically distant from the patient’s bedside round. Pharmacists must be more productive and more efficacious in transmitting input and knowledge to the team independently. Established relationships with the team had positive effect. Teamwork as multidisciplinary and collaboration is amazing. Physician, pharmacist, nurses, and respiratory therapists, infectious diseases and other specialties like cardiology, nephrology, etc., put the input of their expertise. With persistence compassion for medication safety, patient medication profiles have been reviewed by clinical pharmacists through computerized pharmacy system and health information management system. Room discussion planned where whole team sits together and discusses patient management without exposure to the bedside. All medical staff engaged in this activity wearing all personal protective equipment (PPE). Bedside staff and doctors get connected to this discussion group through video conference calls and discuss the matters related to the patients. When needed telephonic communication was arranged and created a WhatsApp group of all health care professionals. It is always made sure that pharmacist can be approached for any information required and all the medication adjustments are done with pharmacist recommendations.

This initiative significantly helped in COVID-19 recovery in terms of providing them safe and effective pharmaceutical care. The number of clinical pharmacist interventions also support this initiative. Another important aspect of event-driven pharmaceutical care is to provide online real-time clinical supports to health care providers working in the battle zone, including front-line physicians, nurses, and pharmacists. Despite resource limitations and restrictions due to pandemic situations, clinical pharmacy lead created several communication ways, available 24 h. A WhatsApp group was created including all the clinical pharmacists and front-line physicians for immediate communication with all at a time and for timely decision-making. Infectious disease (ID) faculty directly involved ID-clinical pharmacist in real-time for pharmacotherapy consultation and quick decision-making after the approval of off-label drugs.

## Response 3: keeping the front-line health care providers updated through drug evaluation and evidence-based guidelines

Presently, there is no defined treatment option for treating COVID-19 and none of the used medicine has been specifically tested for its safety and efficacy for COVID-19 [8]. In the ongoing evolving situation of the COVID-19 pandemic, the need for drugs increased and is very uncertain, especially for those drugs used off-label. In Pakistan where pharmacists are already mitigating drugs shortage by various strategies [9], it requires more dedicated pharmacy support to conduct evidence-based drug evaluations and establishing the guidelines for our population. For example, judicious use of chloroquine phosphate and antivirals for preventing and treating COVID-19 [10], evaluating efficacy and safety of antivirals [11], and glucocorticoids [12] with monitoring throughout. Further assisting front-line physicians for optimal dosing schedules, appropriate routes of administration. Establishing local rational drug use guidelines is always a practical approach to mitigate the drugs-related issues in the local population. In our hospital, clinical lead pharmacists play a vital role with the hospital leads to establish these guidelines. For updating all the health care providers about the use of medications in COVID-19 patients regularly updated through the generalized mail for all. Addressing the drug indications, available dosage form, dosing schedule, suitable solvents, possible routes of administration, associated ADRs, precautions, and conditions requiring dose adjustment, such as pregnancy, lactation, pediatric and elderly patients, renal and hepatic dose adjustments, etc. [13]. In addition, to keep all the front-line pharmacists updated is highly crucial and challenging. Team members share their learnings and aim for the distinctive goal to overcome this challenge. Recent literature and treatment updates are regularly shared to provide the best possible care to the patients. All this activity is done through emails, WhatsApp groups, and online meetings [14].

## Response 4: guidance for pharmacy staff for continuity of services; redesigned shift hours

Health care staff exposure to the hospital during the pandemic of COVID-19 includes the risk of being infected. To continue the services and to keep the front-line pharmacy staff safe and fit to work, we decided to decrease the exposure of staff to hospital and to minimize the cluster. As in the case, if any staff gets COVID-19 infected quarantine is required, thus this initiative of duty hours redesign helped to have enough backup staff to keep the functionality of the department. In addition, due to reduced hospital admissions and clinic visits [4] this redesigned duty hours also reduced the institutional financial burden. In this situation of persistent anxiety, pharmacy lead maintaining the staff morale high through appreciation and showing empathy for hospital staff directly dealing with the patients and appreciating their selflessness. Staff is specifically trained and reinforced to adhere to personal protective equipment use guidelines.

## Response 5: mitigation of drug shortages issues

Drug shortage during the COVID-19 pandemic has affected almost all the countries. Shortage of both prescribed and over-the-counter drugs may develop. Disruption in the local production process and international transport are the main reasons for the short supply in our country. In our pharmacy, we established an early warning system by following the American Society of Health-System Pharmacists (ASHP) guidelines, through ongoing active surveillance conducted by pharmacists and addressing the shortage issues promptly.

## Response 5: reduced medication wastage by implementation of unit dosage system

The pharmacy department has always been an integral section of the hospital for managing and treating the patient. When faced with major public health emergencies from infectious diseases, 2019-nCoV, the concern is to protect staff so that they do not become infected and protect them from becoming vector or carriers. Prevention and control is done in inpatient pharmacy medication distribution within the hospital.

Physicians enter medication orders for inpatient through physician order entry system (CPOE) and pharmacist verifies the orders while sitting in the satellite pharmacy. In our hospital normally medications are supplied for 24 h to the nursing station by practicing modified-unit-dosage-drug-delivery system. Medication carts are prepared by technicians and pharmacist check for accurate dispensing before sending to the wards [15]. Practicing modified 24-h medications supply, in return, increases the number of medications return to pharmacy because of extra dose dispensing of more than one time and new order entry for the admitted patients [16]. Various medications are returned to pharmacy from the ward where they were not used. Before the COVID-19 pandemic, a number of unused medications are return to pharmacy in the form of regular credit that was about 15% of the total medications dispensed. In critical areas after positive cases being treated in hospital, the drugs exposed directly to the environment of the wards of COVID-19 were decided not to be returned for credit to protect the staff [17, 18]. We decided to make changes in the process flow to stop this wastage of expensive medications and ultimately save the cost. Modified unit dosage form (24 h supply) was switched to unit dosage form (one dose of a drug dispensed at a time). As a result, not only the wastage is prevented, but the number of medicine return to pharmacy also decreased and saved the pharmacy staff time previously wasted in rework. Along with the implementation of the actual unit dosage system, we also modified the process and placed drugs cassette in the area entry, only the drugs need to be administered were taken to the isolation ward and all others kept outside so that they can be returned. Dedicated medication staff were assigned to handover the medicine to the assigned staff. Implementing actual unit dose dispensing system is a big challenge in resource-limiting settings, but we accepted and met the goals of reduced wastage and improved safety.

## Discussion and conclusion

SARS-CoV-2 is an extremely transmissible virus, with rapid disease progression. All the front-line healthcare providers nonstop explore the most appropriate prevention, treatment, and diagnostic techniques. In this pandemic situation in Pakistan, a country with limited resources, all the clinical pharmacists under the leadership command collaborated to actively participate and give maximum utility of their pharmacological expertise with activities modification. We conclude that the clinical pharmacist holds the stout position of academic leader for formulating directions and recommendation, and simultaneously a strong practitioner of pharmaceutical services, through the provision of medical advice to front-line healthcare providers and safeguarding the rational drug usage during the pandemic. Equally, we explored that despite limited resources this pandemic situation has driven the leaders of clinical pharmacy in Pakistan to develop innovative and remote pharmaceutical services.

## Data Availability

Not applicable.
